# Evaluation of Serological Tests for Detection of Antibodies against Lumpy Skin Disease Virus

**DOI:** 10.1128/JCM.00348-20

**Published:** 2020-08-24

**Authors:** Nina Krešić, Ivana Šimić, Tomislav Bedeković, Žaklin Acinger-Rogić, Ivana Lojkić

**Affiliations:** a Virology Department, Croatian Veterinary Institute, Zagreb, Croatia; b Ministry of Agriculture, Veterinary and Food Safety Directorate, Zagreb, Croatia; University of Tennessee at Knoxville

**Keywords:** ELISA, lumpy skin disease, serology assays, virus neutralization test

## Abstract

Lumpy skin disease (LSD) is an emerging, transboundary viral pox disease affecting cattle of all ages and breeds. The serological assay for monitoring immunity following vaccination is a virus neutralization test (VNT/OIE) that determines the neutralization index (NI). The first validated enzyme-linked immunosorbent assay (ELISA; IDVet) has become commercially available, facilitating large-scale serosurveillance for LSD. Although the VNT is labor intensive and time consuming, it is still the recommended test by the OIE. Thus, in this study, we modified the virus neutralization test by employing Madin-Darby bovine kidney (MDBK) cells. The qualitative results obtained with the modified method were compared to the qualitative results obtained by VNT/OIE and ELISA. We used blood sera received within a surveillance program for LSD in 2018. In total, 291 serum samples were tested using VNT/MDBK and ELISA. Of 291 samples, 80 samples were tested by VNT/OIE and used for comparison of the performances between VNT/MDBK and VNT/OIE. The compatibility of results obtained by VNT/MDBK and VNT/OIE resulted in a kappa index of 0.9 with overall proportion agreement of 0.96. Agreement between VNT/MDBK and VNT/OIE was achieved in 56 positive and 21 negative samples. The compatibility of results obtained by ELISA and VNT/MDBK were compared on 291 samples in total and resulted in a kappa index 0.834 with overall proportion agreement of 0.955. Agreement between ELISA and VNT/MDBK was achieved in 238 positive and 40 negative samples. The results obtained demonstrated a strong correlation between VNT/MDBK and the other two methods, indicating the suitability of VNT/MDBK for the detection of the LSD virus-specific neutralizing antibodies.

## Abstract (precis)

Lumpy skin disease (LSD) is an emerging, transboundary viral pox disease affecting cattle of all ages and breeds. The serological assay for monitoring immunity following vaccination is a virus neutralization test (VNT/OIE) that determines the neutralization index (NI). The first validated enzyme-linked immunosorbent assay (ELISA; IDVet) has become commercially available, facilitating large-scale serosurveillance for LSD. Although the VNT is labor intensive and time consuming, it is still the recommended test by the OIE.

## INTRODUCTION

Lumpy skin disease (LSD) is a highly contagious transboundary disease in cattle of all ages and breeds (1) characterized by fever, lymphadenopathy, and nodular skin lesions which can be observed also in the mucous membranes of the eyes and in the respiratory and gastrointestinal tracts (2). Until recently, the endemic geographic range of LSD was limited to the African continent, including Madagascar (3). The disease has spread widely throughout the Middle East (4–7) and Balkan regions (8), southern Caucasus, and parts of the Russian Federation (9), attracting huge attention.

The etiological agent is Lumpy skin disease virus (LSDV), a large double-stranded DNA virus classified within the *Capripoxvirus* genus (CaPV), subfamily *Chordopoxvirinae*, family *Poxviridae* (2019 ICTV). Members of the *Capripoxvirus* genus, including sheeppox virus (SPV), goatpox virus (GPV), and LSDV, share 97% nucleotide identity and are serologically cross-protective. LSDV exists in two infectious forms: the mature virions (MVs), which have a single outer membrane, and enveloped virions (EVs), which have an additional membrane, specialized in cell-to-cell spread (10).

The susceptibility of the host to the infection with LSDV is influenced by many factors, and the interaction between the host immune response and the virus determines the outcome. Immunity to CaPV infection is predominantly cell mediated (11), because most progeny viruses remain inside the infected cells. By spreading locally and directly from cell to cell, the virus is out of the reach of circulating antibodies. The extracellular enveloped virions, which are released by budding from infected cells, may infect neighboring cells or escape into the blood and be disseminated throughout the body (11). Transmission of LSDV is achieved mechanically by blood-feeding arthropods such as mosquitoes (Aedes aegypti) (12), stable flies (Stomoxys calcitrans), and ticks (Amblyomma hebraeum and Rhipicephalus appendiculatus) (13, 14).

A natural resistance to LSDV infection is known in cattle, and subclinical LSDV infections are common (11). LSDV causes significant economic losses, mainly by inducing severely reduced milk production, weight loss, abortion, infertility, and hide damage (15). For successful LSD control, vaccination of all susceptible animals is considered to be the main pillar, supported by other control measures such as stamping out, animal movement restrictions, and vector control (16). In vaccinated animals, antibodies appear 10 days postvaccination and reach a peak 30 days later. A local response to the vaccine usually correlates with good antibody production (4). As with infection with virulent wild-type virus, some bovines are refractory to LSD vaccination, failing to develop a local reaction or detectable levels of antibodies (4).

The serological assay for monitoring immunity following vaccination recommended in the OIE terrestrial manual is a virus neutralization test (VNT/OIE) that determines the neutralization index (NI). The first validated enzyme-linked immunosorbent assay (ELISA) (manufactured by IDVet) has become commercially available, facilitating large-scale serosurveillance for LSD (11). This ELISA is able to detect antibodies against capripoxviruses (LSDV, SPV, and GPV) from approximately 20 days until 7 months postvaccination.

In this study, we modified the virus neutralization test by using Madin-Darby bovine kidney (MDBK) cells and compared the performances of the method with the recommended VNT/OIE test and available ELISA. For this purpose, we used blood sera received for a surveillance program for lumpy skin disease in 2018. The cattle population in Croatia was vaccinated with two live vaccines, Lumpyvax, MSD, and Lumpy skin disease vaccine for cattle, Onderstepoort Biological Products, in 2016 and 2017 during the preventive vaccination campaign approved by the European Commission 1 January 2016. In the beginning of the 2018, the preventive vaccination campaign was stopped and a surveillance program for LSD was started, with an aim to monitor immunity in vaccinated cattle (17).

## MATERIALS AND METHODS

### Animal ethics.

Blood samples were taken from privately owned bovines within the frame of the monitoring for LSD ordered by the Ministry of Agriculture, the Veterinary and Food Safety Directorate. The sampling was performed in line with the principles of good veterinary practice and in full respect of animal welfare. Since this study does not include any animal experiments, the Board of Ethics (Croatian Veterinary Institute) decided that no formal approval was required and that the study is in accordance with national legislation (18).

### Blood samples.

Bovine blood samples (*n* = 291) received for the LSD surveillance program in 2018 were used for the evaluation of the VNT on MDBK cells (VNT/MDBK) and its comparison with ELISA (IDVet, France). Of 291 samples, 80 samples were tested by VNT/OIE and used for comparison to VNT/MDBK. Blood samples were centrifuged at 2,000 rpm (939 × *g*) for 10 min. Serum was transferred to 2-ml tubes (Eppendorf, Germany) and stored at −20°C until analysis. Serum samples were inactivated at 56°C for 30 min for VNTs.

### Virus.

LSDV (Neethling vaccine strain) isolated from a skin nodule on a vaccinated animal as previously described (19) was used for VNT tests. In brief, the sample of nodule skin obtained by biopsy was homogenized in Dulbecco’s modified Eagle’s medium (DMEM) with sterile sand and centrifuged at 3,000 rpm (2,100 × *g*). Supernatant was filtrated trough 0.45-μm pores (Merck Millipore, USA). A suspension of 5 × 10^6^ MDBK cells placed in T25 flasks (Nunc, Thermo Fisher Scientific, USA) was infected with 1 ml of supernatant. Flasks containing infected cells were incubated 1 h at 37°C. After the incubation period, the medium composed of 89% DMEM high glucose (Thermo Fisher Scientific, USA) supplemented with 10% fetal bovine serum (FBS) (Thermo Fisher Scientific, USA) plus 1% penicillin-streptomycin-amphotericin B (Sigma-Aldrich, USA) (basal medium) was added and incubated at 37°C. The cells were examined daily for the presence of a viral cytopathic effect (CPE). After 72 h, the flasks were frozen and thawed and the content was centrifuged at 3,000 rpm (2,100 × *g*) for 10 min. The supernatant (5 ml) containing virus was used to infect 8 × 10^6^ cells in suspension in T75 flasks (Nunc, Thermo Fisher Scientific, USA) according to the above-described procedure. Virus was passaged in total eleven times. Aliquots of supernatant containing virus were titrated in 96-well plates according to standard procedures.

### Virus titration.

Virus stock titration was performed in 96-microwell plates (Nunc, Thermo Fisher Scientific, USA). Virus was titrated using 10-fold dilution (10^−1^ to 10^−10^). Two hundred seventy microliters of DMEM high glucose was added in wells in six columns. In six wells in first row, 30 μl of virus stock was added. After thorough mixing with an automatic pipette, 30 μl of first dilution was transferred to the next row. The procedure was repeated until a final dilution of 10^−10^ was reached. One hundred microliters of prepared dilutions was transferred to the corresponding wells of a new 96-microwell plate (Nunc, Thermo Fisher Scientific, USA). Complete medium for MDBK cell cultivation was removed from the T75 flask (Nunc, Thermo Fisher Scientific, USA). The cells were trypsinized, and 100 μl of the cell suspension (10^5^/ml) was added to each well. Four wells were used for cell control and four wells for virus control. The plate was incubated at 37°C with 5% CO_2_ for 72 h and inspected daily for the presence of a cytopathic effect (CPE). Cell control wells had to demonstrate the absence of CPE, and characteristic CPE in the form of lumps on the cell layer had to be present in wells for the virus control. Virus titer was calculated according to the Spearman-Karber method. The results are expressed as decimal logarithms (D_50_).

### Positive- and negative-control sera.

As positive- and negative-control sera, we used national positive and negative controls. National negative-control serum was prepared using serum samples obtained from the animals before vaccination against LSDV during our previous study (15). National positive-control serum was prepared using serum samples obtained from the animals 4 weeks after vaccination against LSDV. Negative- and positive-control serum samples were tested with ELISA and VNT/MDBK.

### ELISA.

Serum samples (*n* = 291) were tested using ID Screen Capripox Double Antigen ELISA (IDVet) for the detection of antibodies against capripox viruses according to the manufacturer’s instructions.

### Virus neutralization test.

Serum samples (*n* = 80) were tested using the VNT procedure prescribed by the OIE terrestrial manual (2017), which determines the neutralization index as the preferred method. The virus strain was titrated against a constant dilution of test serum. In that way, a larger volume of test serum is required, but the difficulty of ensuring 100 for the 50% tissue culture infective dose (TCID_50_) is neutralized. The test was performed in 96-well plates (Nunc, Thermo Fisher Scientific, USA) using lamb testis (LTe) cells according to the available OIE procedure.

### Virus neutralization test on MDBK cells.

Bovine serum samples (*n* = 291) were analyzed using a modified VNT procedure to determine LSDV antibody titer. The test was performed in 96-microwell plates (Nunc, Thermo Fisher Scientific, USA). Each test contains a control plate used for virus back titration and for positive and negative serum titration and a plate for testing serum samples.

For the virus back titrations, 150 μl of DMEM high glucose (Thermo Fisher Scientific, USA) was added to appropriate wells in columns 1 to 4 of the control plate. One hundred microliters of DMEM high glucose (Thermo Fisher Scientific, USA) was added to appropriate wells in columns 5 to 12. Fifty microliters of the working virus was added to four wells in row H (columns 1 to 4), and 4-fold serial dilutions (up to 1/65,536) were made. Fifty microliters of positive serum sample was added to four wells in row H (columns 5 to 8), and 50 μl of negative serum sample was added to four wells in row H (columns 9 to 12). Threefold dilutions were made (one-third up to 1/6,561).

Serum samples to be tested were examined on new 96-microwell plates. One hundred microliters of DMEM high glucose (Thermo Fisher Scientific, USA) was added to each well of the 96-microwell plate for serum sample dilution. Fifty microliters of the examining serum samples was added in duplicate wells in row H, and 3-fold dilutions were made (until 1/6561). It is possible to test 6 samples per plate.

After all dilutions were made, 50 μl of the working virus was added to all wells containing diluted positive, negative, and tested serum samples and in the wells for infection control. The plates were incubated at 37°C with 5% CO_2_ for 1 h. Following the incubation period, 50 μl of MDBK cells (5 × 10^5^/ml) was added and incubated at 37°C with 5% CO_2_ for 72 h. The plates were inspected for the presence of cytopathic effect (CPE). The absence of CPE in wells with a dilution of one-third was considered positive for the presence of antibodies. Titer was calculated according to Spearman-Karber method. The results are expressed as decimal logarithms (D_50_).

### Validation of the VNT/MDBK.

To validate the test, national reference positive serum and back titration of the virus working dilution were titrated in 10 independent trials. The results are expressed as decimal logarithms (D_50_) and used to calculate the means and the standard deviations for the national reference positive serum and working virus. The test was considered valid when the D_50_ of the working virus control and the D_50_s of the negative and positive national reference sera were within one standard deviation.

### Statistical methods.

To determine the specificity and the sensitivity of VNT/MDBK, we used VNT/OIE as the gold standard. We determined true positives (TPs) (VNT/MDBK positive and VNT/OIE positive), false positives (FPs) (VNT/MDBK positive and VNT/OIE negative), false negatives (FNs) (VNT/MDBK negative and VNT/OIE positive), and true negatives (TNs) (VNT/MDBK negative and VNT/OIE negative). The formulae used were TP/(TP + FN) × 100 for the sensitivity and TN/(FP + TN) × 100 for the specificity. The same procedure was used for the specificity and the sensitivity determination of VNT/MDBK cells in comparison to ELISA results. To compare the performance of used tests that were evaluated on the same serum samples originating from vaccinated cattle, the kappa test was used according to http://epitools.ausvet.com.au/content.php?page=Compare2Tests.

## RESULTS

### Virus neutralization test.

Of 80 bovine serum samples, 59 (73.75%) tested positive and 21 (26.25%) tested negative (Table 1). Among positive samples, 24 samples demonstrated an NI of 1.5, 16 samples had an NI of 2, and 19 samples had an NI of 2.5.

**TABLE 1:** Results of the serological tests used for the detection of LSDV specific antibodies

Result	ELISA		VNT/MDBK		VNT/OIE	
	*n*	%	*n*	%	*n*	%
Positive	250	85.91	239	82,13	59	73.75
Negative	41	14.09	52	17,87	21	26.25
Total	291	100	291	100	80	100

### ELISA.

Of 291 bovine serum samples, 250 (85.91%) tested positive and 41 (14.09%) tested negative with the ID Screen Capripox Double Antigen ELISA (Table 1).

### Virus neutralization test on MDBK.

Of 291 bovine serum samples, 239 (82.13%) tested positive and 52 (17.87%) tested negative with VNT/MDBK (Table 1). Among positive samples, 95 had antibody titer log D_50_s of 0.72 to 1.19, 121 samples had titer log D_50_s of 1.43 to 1.9, and 23 samples had titer log D_50_s of 2.14 to 2.39.

### Comparison of the performance of the ELISA, VNT/MDBK, and VNT/OIE.

The performances of VNT/MDBK and ELISA were compared to that of VNT/OIE on 80 samples in total. The highest number of positives was detected by ELISA (*n* = 64), followed by VNT/OIE (*n* = 59) and VNT/MDBK (*n* = 56).

The compatibility of results obtained by VNT/MDBK and VNT/OIE resulted in a kappa index of 0.90 with overall proportion agreement of 0.96. No false positives were detected with VNT/MDBK. Three samples had an NI of 1.5 in VNT/OIE, and in VNT/MDBK, their neutralization activity was detected in 50% of the wells at a dilution of one-third. Since that value was below the chosen cutoff, they were considered negative. Agreement between VNT/MDBK and VNT/OIE was achieved in 56 positive and 21 negative samples. The sensitivity of the VNT/MDBK compared to that if VNT/OIE was 95%, and specificity was 100% (Table 2).

**TABLE 2:** Comparison results for VNT/MDBK, ELISA and VNT/OIE

VNT/MDBK result^*a*^	No. of samples or value (%)			
	ELISA		VNT/OIE^*b*^	
	+	−	+	−
+	238	1	56 (TP)	0 (FP)
−	12	40	3 (FN)	21 (TN)
Sensitivity (%)	95		94.91	
Specificity (%)	97.56		100	

a +, positive; −, negative; sensitivity, TP/(TP + FN) × 100; specificity, TN/(FP + TN) × 100.

b TP, true positive; FP, false positive; FN, false negative; TN, true negative.

The compatibility of results obtained by ELISA and VNT/MDBK was compared on 291 samples in total and resulted in a kappa index of 0.834 with overall proportion agreement of 0.955. Agreement between ELISA and VNT/MDBK was achieved in 238 positive and 40 negative samples (Table 2). In total, 12 positives were detected with ELISA that were VNT/MDBK negative, and one sample that was ELISA negative was VNT/MDBK positive. The sensitivity of VNT/MDBK compared to that of ELISA was 95%, while specificity was 97.56% (Table 2).

## DISCUSSION

We described a modified VNT using MDBK cells and compared it with the current gold standard VNT/OIE and commercially available ELISA. Although VNT is labor intensive and time consuming and requires biosafety level 3 (BSL3) containment in disease-free countries (16), it is still the recommended test in the OIE terrestrial manual.

Our aim was to modify the VNT that measures LSDV antibody titers and to evaluate its performance in comparison with VNT/OIE and commercially available ELISA. The aim was also to adopt a VNT method that overcomes certain difficulties related to the use of LTe cells in VNT/OIE. Specifically, the use of LTe cells in the VNT/OIE makes it more time consuming than the use of MDBK cells in a modified VNT. It was already described that OA3Ts cells exhibit a shallow exponential growth phase until 96 h postseeding (19), resulting in longer expansion time. Furthermore, besides differences in growth kinetics, differences in the morphology of cells with low and high passage numbers were described. Cells with lower passage numbers (p17 to p27) were observed in greater numbers and had a uniform cell morphology, while cell viability at each passage level was determined to be consistently greater than 97% (19). Under our conditions, similar morphological changes and expansion characteristics were observed.

The VNT/OIE test procedure is time consuming, and the incubation period takes up to 9 days. Under our conditions, in VNT/OIE, the first signs of the CPE occurred 72 h postinfection in wells infected with high levels of virus (log_10_ 4 and log_10_ 5). The specific changes were not clearly evident in wells with lower levels of virus (log_10_ 1.5 to log_10_ 3.5) 72 h postinfection (p.i.). The specific CPE became evident 96 h p.i. In VNT/MDBK, the first signs of CPE were evident 48 h p.i., and after 72 h, the specific changes were clearly evident as described also by Samojlović et al. (20). Furthermore, the test results 72 h p.i. are in line with the results observed 96 h p.i. (data not shown). The CPE caused by LSDV in MDBK cells was clearly different from the LSDV CPE in LTe cells. The effects of LSDV on MDBK manifested as cell proliferation and accumulation in the forms of lumps on the cell monolayer, as already demonstrated (20).

Two previous studies (16, 20) also described VNT for LSDV antibody titer estimations. The methods were technically similar to those for the VNT/MDBK used here, but the VNT/MDBK is the only one that compared the results to the gold standard VNT/OIE. Differences related to VNT described by Samojlović et al. (20) are reflected in several factors, such as the virus used, dilution fold, and slight difference in cell number. Samojlović et al. (20) used wild-type virus isolated during an LSD outbreak in Serbia, while we used a vaccine strain isolated from a skin nodule on a vaccinated animal (19), which was passaged eleven times in MDBK cells with clear cytopathic effect. The use of an isolated vaccine strain for VNT proved to be very useful when there are no cases of infection with the wild type in the country. For sample dilutions (2-fold), Samojlović et al. (20) used Eagle MEM with HEPES buffer and with 10% fetal bovine serum (FBS). The use of DMEM high glucose for sample dilution (3-fold), as described here, proved to be satisfying and lowered the amount of FBS used and thus the overall costs of the test. Additional differences observed are related to the cell number used and the performance of 8-well replicates instead of two-well replicates described here. Milovanović et al. (16) described 2-fold dilutions, with initial dilutions of one-tenth, in triplicates, incubated with the Neethling vaccine strain. Milovanovic et al. (16) neither clearly described back titration of the virus nor reported the exact cell number used. The final reading was performed after 7 days by Milovanovic et al. (16) and after 72 h under our conditions. The cutoff value of positivity was one-half in the VNT used by Samojlović et al. (20), one-tenth in the VNT used by Milovanović et al. (16), and one-third in our method. The dilution of one-tenth as the cutoff value might cause low-positive samples to be missed.

The highest number of positives detected by ELISA was not unexpected and was already reported. A mismatch of detected positive and negative cattle was seen to occur between ELISA and VNT in 26 cases in the study by Milovanović et al. (16). The same nonconformity of serological tests was reported by Babiuk et al. (21), which was explained by the detection of different anticapripoxvirus antibodies with the different tests used. The strong correlation between VNT/OIE and VNT/MDBK indicates the suitability of VNT/MDBK for the detection of the LSDV-specific neutralizing antibodies. We are aware that the results would be more confident if the comparison included a larger sample size. Three samples tested by VNT/OIE expressed values at the limit of positivity (NI, 1.5). The neutralization activity of those samples was also detected by VNT/MDBK in 50% of the wells. Since the detected neutralization activity occurred below the chosen cutoff value, those samples were considered negative. In addition, the antibody level following vaccination can be low or below the limits of detection of serological methods. As already stated by Samojlović et al. (20), the virus neutralization test is considered to be the most specific serological method, but it is not sensitive enough to detect antibodies in each animal that has been in contact with the virus. Furthermore, when assessing the results of serological tests for LSD diagnostics, some specifics of immune responses to LSDV infection must be kept in mind. The immunity is predominantly cell mediated, and vaccinated animals or those showing mild disease may develop only low levels of neutralizing antibodies, which are often below the detection limits of currently available serological tests (11). The vaccinated animals do not mount an antibody response following virulent challenge, supporting the role of cell-mediated immunity in protection from capripoxvirus disease (16). Despite those immunological specificities, serological assays are recommended as suitable methods to investigate relatively recent outbreaks and can be used to demonstrate the disease-free status of a country provided that testing is carried out at regular intervals. In addition to VNT as the gold standard, immunoperoxidase monolayer assays (IPMAs) and indirect fluorescent antibody tests (IFATs) can be used for serological surveys. Although VNT is labor and time consuming, it can be modified slightly to increase the number of samples tested on one plate and to reduce the time to read the results (22).

The current knowledge on the time span of antibody detection after vaccination is heterogeneous (16). A significant increase of capripoxvirus-specific antibody titers is described to be seen from day 21 up to day 42 after vaccination, and antibodies remain detectable for approximately 7 months. An immunological study conducted in cattle after vaccination with LSDV showed that detection of specific antibodies is limited to 40 weeks postvaccination (23), while Milovanović et al. (16) reported the detection of antibodies up to 46 to 47 weeks after vaccination. The maximum duration of protection has been reported to be 22 months, and the immune status of a previously infected or vaccinated animal cannot be related directly to serum levels of neutralizing antibodies. Antibodies against CaPV can usually be detected for 3 to 6 months after infection, but further studies are required to investigate the long-term persistence of CaPV antibodies postinfection (11).

### Conclusions.

Serological assays are recommended as suitable methods to investigate relatively recent outbreaks and can be used to demonstrate the disease-free status of a country provided that testing is carried out at regular intervals. Results obtained with VNT/MDBK demonstrated a strong correlation to those of VNT/OIE and ELISA, which indicates its suitability for LSDV neutralizing antibody detection.
